# Draft Genome Sequences of Multidrug-Resistant Escherichia coli Isolated from River Water

**DOI:** 10.1128/mra.00827-22

**Published:** 2022-10-12

**Authors:** Issmat I. Kassem, Jouman Hassan, Malak A. Esseili, David Mann, Marwan Osman, Shaoting Li, Xiangyu Deng

**Affiliations:** a Center for Food Safety, Department of Food Science and Technology, University of Georgia, Griffin, Georgia, USA; b Department of Public and Ecosystem Health, College of Veterinary Medicine, Cornell University, Ithaca, New York, USA; c Cornell Atkinson Center for Sustainability, Cornell University, Ithaca, NY 14853, USA; University of Delaware

## Abstract

The spread of antibiotic resistance poses a critical challenge worldwide. Contaminated environments can become reservoirs, spreading antibiotic-resistant bacteria and genetic determinants of resistance to humans directly or indirectly. Here, we report the draft genome sequence, the resistome, virulence genes, and sequence types of seven multidrug-resistant Escherichia coli strains isolated from river water.

## ANNOUNCEMENT

Surface waters in Lebanon have been threatened by pollution (1–4). Previously, we showed that up to ~74% of water samples from rivers in Lebanon exceeded the microbiological acceptability standards for irrigation due to the presence of fecal indicators (1). Additionally, 45.8% of the Escherichia coli strains isolated from these rivers were classified as multidrug resistant (MDR) (1). Here, we selected 7 MDR E. coli strains isolated from different rivers for whole-genome sequencing (WGS) analysis.

Composite water samples (1 L) were collected from rivers in Lebanon (Table 1). The water samples were filtered using 0.22-μm Millipore membranes that were placed onto RAPID'E. coli 2 agar plates (Bio-Rad, USA) (1, 4). The plates were incubated at 37°C under aerobic conditions for 24 h, and colonies that showed an E. coli phenotype (violet-to-pink color) were selected, purified, and further identified using a species-specific PCR analysis (1–3).

**TABLE 1:** Genome properties and antibiotic resistance profiles of the E. coli isolated from river water in Lebanon

River	Location	Isolate ID^*a*^	Genome size (bp)	No. of reads	No. of contigs	*N*_50_ (bp)	*L*_50_	Genome coverage (×)	GC content (%)	Antibiotic resistance phenotype^*b*^	Acquired antibiotic resistance genes (mutations)^*c*^	Antimicrobial resistance genes^*d*^	Virulence genes	Human pathogen^*e*^ (probability)	Sequence type	Detected plasmid types	GenBank accession no. of assembled genomes
Al-Kabir	Wadi-Khaled	A10	4,974,255	638,984	345	41,111	38	37.90	51.1	R: AMP-AMC-CTX-LEX-CFM-STR-TET-SXT-CHL; IR: GEN-KAN; S: FEP-DOR-IPM-MEM-CIP-NOR	(*gyrA*; p.S83L)	*acrA*; *msbA*; *yojI*; *acrD*; *eptA*; *mdtN*; *mdtO*; *mdtP*; *mdtG*; *mdtH*; *baeS*; *hns*; *acrF*; *acrE*; *ampC*; *marA*; *cpxA*; *tolC*; *bacA*; *mdtE*; *mdtF*; *kdpE*; *emrR*; *emrA*; *emrB*; *emrK*; *evgA*	*cea*; *fyuA*; *gad*; *irp2*; *iss*; *iutA*; *kpsE*; *sitA*; *terC*; *traT*	Yes (0.926)	10	Col(BS512); Col8282; ColpVC; IncX4	JAFJVZ000000000
	Hekr el Dahri	A6	4,854,745	642,928	441	77,125	18	38.15	51.4	R: AMP-AMC-FEP-CTX-LEX-CFM-STR-TET-SXT; IR: IPM; S: DOR-MEM-GEN-KAN-CIP-NOR-CHL	*aph3''-Ib*; *aph6-Id*; *bla*_CTX-M-15_; *bla*_DHA-1.1_; *bla*_TEM-1B_; *dfrA14*; *dfrA17*; *mphA*; *qnrB4*; *qnrS1*; *sul1*; *sul2*; *tetA*	*evgS*; *evgA*; *emrK*; *msbA*; *bacA*; *tolC*; *acrB*; *acrA*; *mdtN*; *acrE*; *acrS*; *mdtG*; *mdtH*; *hns*; *marA*; *emrB*; *emrR*; *mdtE*; *qnrB4*; *bla*_DHA-1_; *mphA*; *bla*_TEM-1_; *bla*_CTX-M-15_; *sul2*; *dfrA14*	*gad*; *iss*; *ompT*; *sitA*; *terC*; *traT*	Yes (0.925)	540	Col(MG828); Col156; IncFIB; IncFII	JAFJVU000000000
Assi	Bejaj	A7	5,312,718	835,855	385	62,299	27	48.90	51.4	R: AMP-AMC-CTX-LEX-GEN-KAN-STR-TET-CIP-NOR-SXT-CHL; IR: FEP-CFM-DOR-IPM; S: MEM	(*pmrB*; p.V161G)	*tolC*; *pmrF*; *msbA*; *mdtH*; *mdtG*; *mdtE*; *marA*; *kdpE*; *hns*; *evgA*; *acrA*; *emrR*; *emrB*; *cpxA*; *baeS*; *acrE*; *acrD*; *acrB*	*gad*; *lpfA*; *ompT*; *papC*; *terC*	Yes (0.932)	10772	IncFIB; IncFII; IncX4	JAFJVT000000000
Ibrahim	Afqa	RB3	4,675,206	436,525	171	128,766	11	41.61	51.3	R: AMP-AMC-STR-TET-SXT; IR: KAN; S: FEP-CTX-LEX-CFM-DOR-IPM-MEM-GEN-CIP-NOR-CHL	No hits with known phenotypes in the database using default parameters	*acrB*; *acrA*; *marA*; *mdtE*; *hns*; *evgA*; *emrY*; *emrR*; *emrB*; *mdtB*; *pmrF*; *acrE*; *cpxA*; *tolC*; *mdtG*; *msbA*	*celb*; *gad*; *lpfA*; *ompT*; *terC*	Yes (0.943)	2766	Col156	JAFJXE000000000
Beirut	Beirut Port	RB8	5,164,606	442,592	239	90,327	16	41.34	51.6	R: CFM-DOR-IPM-MEM-GEN-CIP-NOR-CHL; IR: AMP; S: AMC-FEP-CTX-LEX-KAN-STR-TET-SXT	*aph3”-Ib*; *aph6-Id*; *sul2*; *tetB*	*emrK*; *evgA*; *marA*; *mdtG*; *mdtH*; *cpxA*; *emrR*; *emrB*; *baeS*; *tolC*; *hns*; *msbA*; *mdtF*; *sul2*	*air*; *chuA*; *eilA*; *etsC*; *fyuA*; *gad*; *hlyF*; *ireA*; *iss*; *mchF*; *papA_F12*; *irp2*; *papC*; *sitA*; *terC*; *traT*	Yes (0.936)	38	IncFIA; IncFIB; IncFII	JAFJXA000000000
Awali	Barouk	A2	5,303,506	694,775	308	103,419	16	41.07	51.2	R: AMP-AMC-FEP-CTX-LEX-CFM-DOR-MEM-GEN-KAN-STR-TET-CIP-NOR-SXT-CHL; IR: IPM	*aph3”-Ib*; *aph6-Id*; *bla_T_*_EM-1B_; *dfrA5*; *sul2*; *tetA*	*marA*; *acrB*; *acrA*; *mdtG*; *mdtH*; *baeR*; *mdtA*; *tolC*; *mdtM*; *msbA*; *evgA*; *acrE*; *hns*; *cpxA*; *emrR*; *emrB*; *dfrA5*; *bla*_TEM-1_	*afaA*; *afaB*; *afaD*; *afaE8*; *cia*; *cvaC*; *etsC*; *f17A*; *f17G*; *fyuA*; *gad*; *hlyF*; *hra*; *iroN*; *irp2*; *iss*; *iucC*; *iutA*; *lpfA*; *mchB*; *mchC*; *mchF*; *mcmA*; *ompT*; *papA_F12*; *papC*; *sitA*; *terC*; *traT*	Yes (0.935)	58	Col(MG828); IncFIB; IncFII; IncQ1	JAFJVW000000000
Damour	Nabaa al Safaa	A14	5,183,133	1,212,229	192	153,762	14	71.87	51.3	R: AMC-FEP-CTX-LEX-CFM-GEN-KAN-STR-TET-CIP-NOR-SXT; IR: AMP; S: DOR-IPM-MEM- CHL	*aadA5*; *aph3''-Ib*; *aph6-Id*; *dfrA17*; *mphA*; *sul1*; *sul2*; *tetA* (*gyrA*; p.S83L)	*baeR*; *gadW*; *mdtH*; *cpxA*; *hns*; *evgA*; *mphA*; *sul1*; *qacEdelta1*; *aadA5*; *dfrA17*; *sul2*	*chuA*; *fyuA*; *gad*; *iha*; *irp2*; *iss*; *iucC*; *iutA*; *kpsE*; *kpsMII*; *ompT*; *papA_F43*; *sat*; *senB*; *sitA*; *terC*; *traT*; *usp*; *yfcV*	Yes (0.932)	131	Col(MG828); Col156; IncFIA; IncFIB; IncFII	JAFJVX000000000

a ID, identifier.

b Resistance to antibiotics was determined using the disk diffusion assay and the Clinical and Laboratory Standards Institute (CLSI) guidelines (17). R, resistance; IR, intermediate resistance; S, susceptibility; AMP, ampicillin; AMC, amoxicillin plus clavulanic acid; FEP, cefepime; CTX, cefotaxime; LEX, cephalexin; CFM, cefixime; IPM, imipenem; DOR, doripenem; MEM, meropenem; GEN, gentamicin; KAN, kanamycin; STR, streptomycin; TET, tetracycline; CIP, ciprofloxacin; NOR, norfloxacin; SXT, trimethoprim-sulfamethoxazole; CHL, chloramphenicol. The antibiotics in the resistance profiles are arranged according to the order of antibiotics/classes listed in the CLSI guidelines.

c Gene mutations associated with antibiotic resistance are included in parentheses. Genes and mutations were detected by ResFinder analysis.

d Only “perfect” hits were reported in the table using CARD’s Antibiotic Resistance Ontology (ARO) terms.

e Predicted probability of being a pathogen is included in parentheses.

Before DNA isolation, the E. coli strains were cultured on RAPID'E. coli 2 agar as described above. The genomic DNA from the E. coli was then isolated and quantified using the QiaAmp DNA minikit (Qiagen, USA) and the Qubit double-stranded DNA (dsDNA) broad-range (BR) assay kit (Invitrogen, USA), respectively, as described in the manufacturers’ protocols (5, 6). The Nextera XT DNA library preparation kit and the Qubit dsDNA high-sensitivity (HS) assay kit (Invitrogen, USA) were used to prepare and determine the concentrations of the sample libraries, respectively (7). The libraries were diluted and denatured according to the Illumina “Denature and Dilute Libraries Guide” protocol A (https://support.illumina.com/content/dam/illumina-support/documents/documentation/system_documentation/miseq/miseq-denature-dilute-libraries-guide-15039740-10.pdf) and loaded into the MiSeq reagent cartridge (MiSeq reagent kit v2, 300 cycles) (7). Sequencing was performed using the paired-end sequencing strategy (2 × 250 bp) with a MiSeq sequencer (Illumina). Low-quality reads were removed with Trimmomatic v0.36 (8). The leading three and the trailing three nucleotides were removed from the reads, and a four-nucleotide sliding window was used to also remove nucleotides from the 3′ ends when the average Phred score dropped below 20. Reads shorter than 75 bp were discarded. Draft genome sequences were assembled using the “–careful” option in SPAdes v3.9.0 (9). Contigs shorter than 200 bp were discarded, and the quality of the draft genomes was evaluated with QUAST v4.5 (10). Sequence types (STs) were determined using the PubMLST database (https://pubmlst.org/) with the MLST software v2.16.2 (https://github.com/tseemann/mlst) (11). The resistome was determined using the Resistance Gene Identifier of CARD (RGI 5.2.1, CARD 3.2.4) and the ResFinder v4.1 database (12, 13). The probability of being a human pathogen, virulence genes, and Inc plasmid types were identified using PathogenFinder v1.1, VirulenceFinder v2.0, and PlasmidFinder v2.1, respectively (14–16). Default parameters were used for all software unless otherwise specified.

The properties of the draft genome sequences are listed in Table 1. All the E. coli strains were predicted to be pathogens and carried genetic determinants that are associated with resistance to important classes of antibiotics (Table 1). The strains belonged to different STs and harbored at least one plasmid type, indicating that contaminated rivers can become sinks for diverse MDR strains and resistance determinants.

The draft genome sequences are important for highlighting the role of contaminated environmental niches, including rivers, in the dissemination of antibiotic resistance.

### Data availability.

The raw sequences for the analyzed strains can be found under accession numbers SRX7741090, SRX7741086, SRX7741087, SRX9744115, SRX9744120, SRX7741048, and SRX7741052. The assembled genome sequences were deposited in GenBank under accession numbers JAFJVZ000000000, JAFJVU000000000, JAFJVT000000000, JAFJXE000000000, JAFJXA000000000, JAFJVW000000000, and JAFJVX000000000.
